# The Expression Patterns of Cytochrome Oxidase and Immediate-Early Genes Show Absence of Ocular Dominance Columns in the Striate Cortex of Squirrel Monkeys Following Monocular Inactivation

**DOI:** 10.3389/fnana.2021.751810

**Published:** 2021-10-13

**Authors:** Shuiyu Li, Songping Yao, Qiuying Zhou, Toru Takahata

**Affiliations:** ^1^Key Laboratory for Biomedical Engineering of Ministry of Education, College of Biomedical Engineering and Instrument Science, Zhejiang University, Hangzhou, China; ^2^Interdisciplinary Institute of Neuroscience and Technology, Zhejiang University School of Medicine, Hangzhou, China; ^3^Department of Neurology of the Second Affiliated Hospital, Zhejiang University School of Medicine, Hangzhou, China

**Keywords:** lateral geniculate nucleus, *Saimiri sciureus*, activity-dependent gene expression, CO blob, vesicular glutamate transporter 2, New World monkeys

## Abstract

Because at least some squirrel monkeys lack ocular dominance columns (ODCs) in the striate cortex (V1) that are detectable by cytochrome oxidase (CO) histochemistry, the functional importance of ODCs on stereoscopic 3-D vision has been questioned. However, conventional CO histochemistry or trans-synaptic tracer study has limited capacity to reveal cortical functional architecture, whereas the expression of immediate-early genes (IEGs), *c-FOS* and *ZIF268*, is more directly responsive to neuronal activity of cortical neurons to demonstrate ocular dominance (OD)-related domains in V1 following monocular inactivation. Thus, we wondered whether IEG expression would reveal ODCs in the squirrel monkey V1. In this study, we first examined CO histochemistry in V1 of five squirrel monkeys that were subjected to monocular enucleation or tetrodotoxin (TTX) treatment to address whether there is substantial cross-individual variation as reported previously. Then, we examined the IEG expression of the same V1 tissue to address whether OD-related domains are revealed. As a result, staining patterns of CO histochemistry were relatively homogeneous throughout layer 4 of V1. IEG expression was also moderate and homogeneous throughout layer 4 of V1 in all cases. On the other hand, the IEG expression was patchy in accordance with CO blobs outside layer 4, particularly in infragranular layers, although they may not directly represent OD clusters. Squirrel monkeys remain an exceptional species among anthropoid primates with regard to OD organization, and thus are potentially good subjects to study the development and function of ODCs.

## Introduction

Inputs from the right and left eyes are segregated into ocular dominance columns (ODCs) in the striate cortex (V1) of many primate species, including macaques and humans (Hubel and Wiesel, 1977; Horton and Hocking, 1998a). The functional importance of ODCs has been controversial since the paradoxical appearance of ODCs was reported in squirrel monkeys (Horton and Adams, 2005). According to Adams and Horton, some squirrel monkeys possess ODCs similar to macaques, whereas others are totally lacking of ODCs (Adams and Horton, 2003). A more bizarre observation was that a portion of V1 of a squirrel monkey showed clear ODCs whereas the rest of V1 did not show any evidence of ODC. On the other hand, it has been reported that V1 neurons in a squirrel monkey appeared capable of stereoscopic depth perception (Livingstone et al., 1995), posing a question about the functional significance of ODCs to stereopsis.

Their ODCs have only been studied using intravitreal injections of a transneuronal tracer or cytochrome oxidase (CO) histochemistry combined with monocular inactivation (MI) (Hendrickson et al., 1978; Hendrickson and Tigges, 1985; Adams and Horton, 2003), but not using other more modern methods. We have recently suggested that CO histochemistry can only reveal activity of geniculo-striate axon terminals or dendrites of cortical neurons that make direct contact with geniculo-striate axon terminals, but does not reveal changes in overall activity of cortical neurons (Takahata, 2016; Yao et al., 2021). On the other hand, the expression levels of *c-FOS* and *ZIF268*, immediate-early genes (IEGs), are estimated to more directly reflect spiking activity levels of neurons occurring 30–60 min before fixation (Herrera and Robertson, 1996; Zangenehpour and Chaudhuri, 2002). We have examined the expression of IEGs in the visual cortex of macaques following MI treatment, and successfully demonstrated more detailed structures related to ODCs or binocular vision (Takahata et al., 2009). Using IEG methods, we have also revealed ODCs in the visual cortex of New World owl monkeys after MI (Takahata et al., 2014). Similarly, ODCs have been clearly revealed by examining activity-dependent gene expression in adult marmosets (Chappert-Piquemal et al., 2001; Nakagami et al., 2013), even though findings of a previous trans-synaptic tracer study suggested that ODCs disappear in the course of neonatal development in marmosets (Spatz, 1989). We have even revealed ODC-like patches in V1 of Long-Evans rats (Laing et al., 2015). In tree shrews, it has been suggested that ocular dominance is segregated into layers rather than columns in V1. Consistent with these reports, our IEG method also revealed layer segregation of ocular dominance in tree shrews, but in greater detail about lamination patterns than previously known (Takahata and Kaas, 2017).

Given our success for revealing ODCs with our IEG method, we applied it to V1 of squirrel monkeys to see whether ODCs and/or unknown brain structures related to ocular dominance can be revealed. We first examined the lateral geniculate nucleus (LGN) to confirm the effects of MI. We then examined CO histochemistry in V1 to see whether individual variation in structure is observed as reported previously. Finally, we examined IEG expression in V1 to address whether ocular dominance-related structure can be detected.

## Materials and Methods

### Animals

The brains of two male and three female adult squirrel monkeys (*Saimiri sciureus*, body weight range of 0.5 to 1.0 kg) were used in this study. The information about each monkey is summarized in Table 1. Three of them were subjected to monocular enucleation. Under ketamine (5–20 mg/kg, i.m.) and medetomidine (0.02–0.08 mg/kg, i.m.) anesthesia, the left eye was enucleated using surgical scissors and forceps. The scar was pressed with stanching foam until bleeding stopped, then the eyelid was sutured. The remaining two monkeys were subjected to MI treatment by intravitreous tetrodotoxin (TTX) injection. Under ketamine (5–20 mg/kg, i.m.) and medetomidine (0.02–0.08 mg/kg, i.m.) anesthesia, 3–5 mL of TTX (1 mM) was slowly injected (~1.0 mL/min) into the vitreous cavity of the left eye through a Hamilton syringe. After eye enucleation or TTX injection, the animals were treated with atipamezole (0.002–0.008 mg/kg, i.m.) for quick arousal and returned to their home cages, where they recovered from anesthesia and could move freely. Except the first case (ID 18-27), the animals were kept in the dark overnight, then exposed to their normal light environment for 1–2 h before perfusion for the purpose of greater induction of IEG expression. Some of the data from these monkeys have already been published for a separate study (Yao et al., 2021).

**Table 1:** Summary of monkeys used in this study.

**Squirrel Monkey ID**	**Gender**	**Body Weight (kg)**	**TTX injection**	**Eye removal**	**Duration between monocular inactivation to perfusion (days)**	**Dark rearing**	**Light stimuli after dark rearing (h)**	**PFA concentration for fixation (%)**
18-27	Female	0.5	Left eye-5ul	None	2	None	Not applicable	1
18-30	Male	1	None	Left eye	14	One night	1.5	4
18-31	Female	0.8	None	Left eye	30	One night	1	4
19-03	Male	0.9	None	Left eye	20	One night	2	2
19-04	Female	0.7	Left eye-3ul	None	2	One night	1	2

### Tissue Preparation

The animals were given an overdose of sodium pentobarbital (>50 mg/kg body weight) and perfused with a sucrose solution [8.5% sucrose, 5 mM MgCl_2_ in 20 mM phosphate buffer (PB)], followed by 1.0–4.0% paraformaldehyde (PFA) in 0.1 M PB. The brain was removed from the skull, and the visual cortex was separated from the rest of the brain and flattened immediately. The flattened visual cortices were immersed in a post-fixative (30% sucrose/4% PFA in PB) at 4°C overnight and cut tangentially at 40 mm using a freezing microtome. The thalamic tissue was immersed in 30% sucrose in PB at 4°C for ~1 week until the tissue sank to the bottom of the container. Then the brain tissue was stored at −80°C or cut coronally at 40 mm using a freezing microtome. All sections were stored at −20°C in a cryoprotectant solution [30% ethylene glycol, 30% glycerol and 40% phosphate-buffered saline (PBS)] until used.

### CO Histochemistry

CO histochemistry was conducted as described previously (Wong-Riley, 1979) with slight modifications. The free-floating sections were washed twice in 5% sucrose in PBS for 5–10 min before immersion in the CO reaction solution. This solution was a mixture of 200 μg/mL cytochrome C (Sigma-Aldrich, St. Louis, MO), 150 μg/mL catalase (Sigma-Aldrich), and 100 μg/mL 3,3'-diaminobenzidine (DAB; Sigma-Aldrich) in 5% sucrose and PBS. Tissues were incubated in this solution for 8–24 h at 37°C while rotating at 30 rpm. Subsequently, these sections were washed three times with PBS, mounted on glass microscope slides, and air-dried. The sections were then dehydrated through a series of increasing ethanol concentrations followed by xylene, and permanently cover-slipped with xylene-based glue. The mounting/dehydration/cover-slipping procedure was the same after other staining procedures as well.

### Nissl Staining

Free-floating sections were post-fixed in 4% PFA for a minimum of 12 h at 4°C. After washing in 0.1 M PB, the sections were mounted on glass microscope slides, and air-dried for several days before staining. The slides with sections were then rinsed successively in deionized water, 90, and 75% ethanol. The sections were stained with 0.1% cresyl violet solution for 5 to 10 min. Then the sections were washed with 0.8% acetic anhydrate in 90% ethanol for 5 to 10 min to remove excess cresyl violet before cover-slipping.

### *In situ* Hybridization (ISH)

To prepare the squirrel monkey-specific probes, a part of each of three genes was cloned using RT-PCR with cDNA prepared from an enucleated eye from one of the squirrel monkeys. The primer sequences for *VGLUT2* were GGCAAGGTCATCAAGGAGAA (forward) and GCACAAGAATGCCAGCTAAAG (reverse) that targeted the 322-713 region of NM_020346 (human *SLC17A6*). The primer sequences for *ZIF268* were CCCAGGACAATTGAAATTTGCT (forward) and AAGGCACCAAGACGTGAAAC (reverse) that targeted the 1878-2678 region of NM_001964 (human *EGR-1*). The primers for *c-FOS* were TGAGCCCTTTGATGACTTCC (forward) and ACTCCATGCGTTTTGCTACA (reverse) that targeted the 970-1537 region of XM_001098940 (macaque *FOS*). The PCR amplicon was purified and inserted into a plasmid vector, pCR™II-TOPO® vector, Dual Promoter (Invitrogen, Waltham, MA) using conventional TA cloning, and amplified in competent cells. The plasmids were harvested and purified with QIAGEN® Plasmid Midi Kit (Qiagen, Hilden, Germany) according to the manufacturer's instructions. For colorimetric ISH, digoxigenin (DIG)-labeled antisense and sense riboprobes were prepared from the plasmids using a DIG-dUTP labeling kit (Roche Diagnostics). ISH with sense probes showed no signals higher than background level.

Our ISH staining protocol was described in our previous papers (Takahata et al., 2014). Briefly, free-floating brain sections were immersed in 4% PFA in 0.1 M PB (pH 7.4) for 1 to 2 days at 4°C until processing. ISH processing began with immersion with 0.3% Triton-X, then treatment with 1–10 mg/mL proteinase K for 30 min at 37°C. After acetylation, the sections were incubated in the hybridization buffer [5x standard saline citrate (SSC: 150 mM NaCl, 15 mM sodium citrate, pH 7.0), 50% formamide, 2% blocking reagent (Roche Diagnostics), 0.1% N-lauroylsarcosine (NLS), 0.1% sodium dodecyl sulfate (SDS), 20 mM maleic acid buffer; pH 7.5] containing 1.0 mg/ml DIG-labeled riboprobe overnight at 60°C. Hybridized sections were washed twice, 20 min each in wash buffer (2x SSC, 50% formamide, 0.1% NLS) at 60°C. Subsequently, the sections were successively immersed in RNase A buffer [10 mM Tris-HCl, 10 mM ethylenediamine-N, N, N', N'-tetraacetic acid (EDTA), 500 mM NaCl, pH 8.0] that contained 20 mg/mL RNase A for 30 min at 37°C, 2x SSC/0.1% NLS for 15 min at 37°C, and 0.2x SSC/0.1% NLS for 15 min at 37°C. Hybridization signals were visualized using alkaline phosphatase (AP) immunohistochemical staining and a DIG detection kit (Roche Diagnostics) that used an overnight reaction to nitro blue tetrazolium chloride/5-bromo-4-chloro-3-indolyl phosphate, toluidine salt (NBT/BCIP) (Roche Diagnostics).

### Data Analysis

The sections were scanned with a VS-120 automated bright field microscope (Olympus, Tokyo, Japan). The images were edited using Adobe Photoshop software (cc 2018, Adobe, San Jose, CA). Images of several different tangential sections were digitally stitched to create a mosaic image of a single layer under the guidance of vasculature patterns, because tangential sections were sometimes comprised of different layers due to incomplete flattening. Brightness and contrast were enhanced and some annotations and nomenclature were added. Some inconsistency exists in the literature concerning the numbering of V1 layers (Balaram et al., 2014). In this study, we used Hässler's scheme for V1 layering (Hässler, 1967) and provided Brodmann's layers in parenthesis.

## Results

### Activity Changes in LGN Neurons Following MI

**Figure 1:**
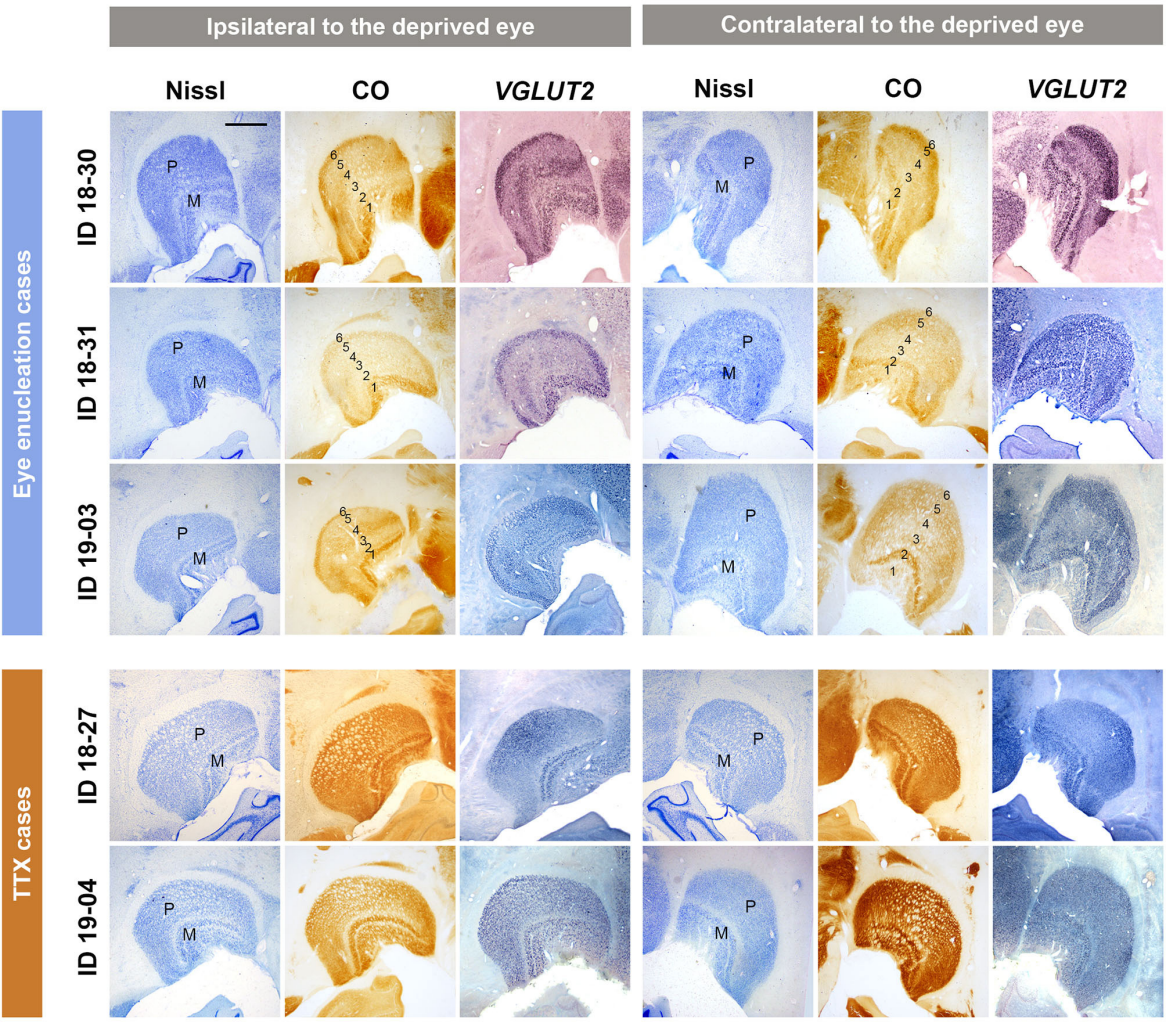
Coronal sections of the LGNs (ID 18-30, ID 18-31, ID 19-03, ID 18-27, and ID 19-04) stained for Nissl substance, CO, and *VGLUT2* mRNA, as annotated. The left panels show the LGNs ipsilateral to the deprived eye, and their left is lateral and upper is dorsal. The right panels show the LGNs contralateral to the deprived eye, and their left is medial and upper is dorsal. The sublayers [parvocellular (P) or magnocellular (M) layers] of LGN are identified by their morphology with Nissl staining. In monocular enucleation cases (ID 18-30, ID 18-31, and ID 19-03), both CO and *VGLUT2* mRNA expression show lower expression levels in LGN layers that receive exclusive inputs from the enucleated eye (layers 2, 3, and 5 for the ipsilateral LGN and layers 1, 4, and 6 for the contralateral LGN) compared to that in LGN layers that receive exclusive inputs from the other eye (layers 1, 4, and 6 for the ipsilateral LGN and layers 2, 3, and 5 for the contralateral LGN), making their 6 laminar structure clear. In monocular TTX injection cases (ID 18-27 and ID 19-04), decreased expression of CO and *VGLUT2* mRNA is not observed. Scale bar = 1 mm.

To confirm activity changes of the inactivated eye pathway following MI treatment, the LGNs were examined for Nissl substance, CO activity, and activity-dependent *VGLUT2* mRNA expression in all cases (Figure 1). As shown in previous studies (Fitzpatrick et al., 1983; Horton and Hocking, 1996), two magnocellular (M) layers were obvious in Nissl staining of ventral medial parts of squirrel monkey LGNs. Parvocellular layers were seen in dorsal lateral parts, but lamination was not evident in Nissl staining, due to the lack of septa or intercalated koniocellular (K) layers in this species, as shown previously. This Nissl staining pattern was not different between the two hemispheres, indicating no change in cellular volume or numbers due to MI treatment. In CO histochemistry, clear laminar differences were observed between hemispheres in monocular enucleation cases (ID 18-30, ID 18-31, and ID 19-03). LGN layers 1 to 6 from ventromedial to dorsolateral were discernable: LGN layers 1 and 2 are for M layers and 3–6 are for P layers, and LGN layers 1, 4, and 6 should exclusively receive projections from the contralateral eye and LGN layers 2, 3, and 5 should exclusively receive projections from the ipsilateral eye (Fitzpatrick et al., 1983; Horton and Hocking, 1996). As anticipated, CO expression was lower in LGN layers that received inputs from the enucleated eye, compared to expression in LGN layers that received inputs from the intact eye in these animals. Since we have previously shown a decrease of *VGLUT2* mRNA expression in the specific LGN layers in macaques that had a monocular retinal lesion (Takahata et al., 2018), we expected to see a decrease of *VGLUT2* mRNA in the LGNs of the squirrel monkeys in this study. As expected, lower levels of *VGLUT2* mRNA signal were observed in LGN layers that receive inputs from the enucleated eye. These results confirm that neuronal activity was significantly decreased in the domains dominant for the enucleated eye without neuronal loss. On the other hand, cross-laminar differences or cross-hemisphere differences were not observed in CO histochemistry or *VGLUT2* ISH in the monocular TTX injection cases (ID 18-27 and ID 19-04), suggesting that the influence of TTX injection was rather moderate, perhaps because of the short inactivation period (2–4 weeks for eye enucleation vs. 2 days for TTX) or incomplete inactivation, even though pupil dilation was observed immediately after TTX injection.

### Overall CO and IEG Expression Patterns in Layer 4 (4C) of V1

**Figure 2:**
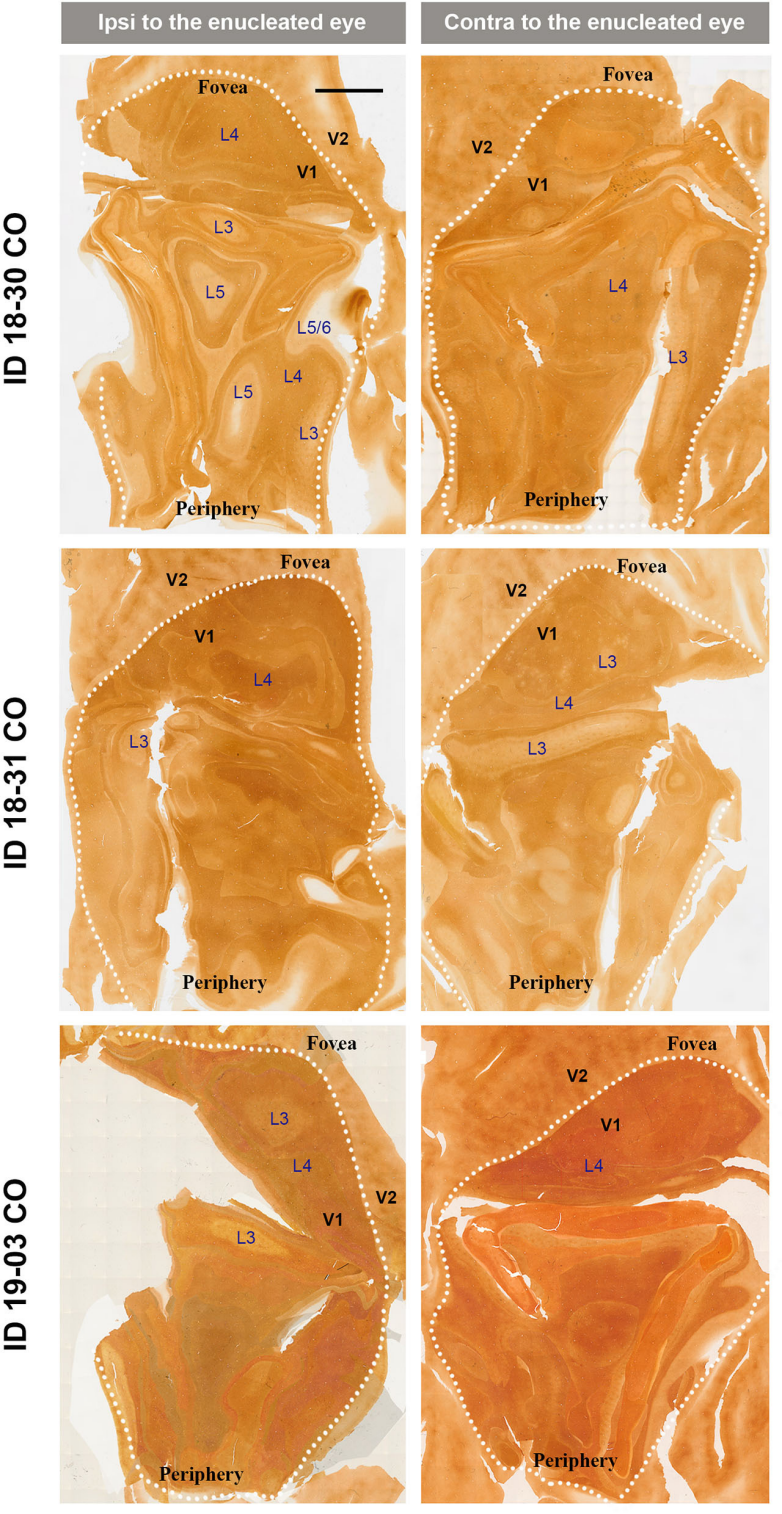
Mosaic images of tangential sections of V1 of eye enucleation cases (ID 18-30, ID 18-31, and ID 19-03) stained for CO activity. The left panels show the ipsilateral, and the right panels show the contralateral hemispheres to the enucleated eye. It is intended to exhibit layer 4 (4C), but because the flattening of V1 is not complete in large sections, other layers of V1 are also included in each image, illustrated as L3–L6. White dashed lines delineate borders between V1 and V2. V1 regions representing the foveal and peripheral portions of the visual fields are indicated as “Fovea” and “Periphery,” respectively. Scale bar = 5 mm.

**Figure 3:**
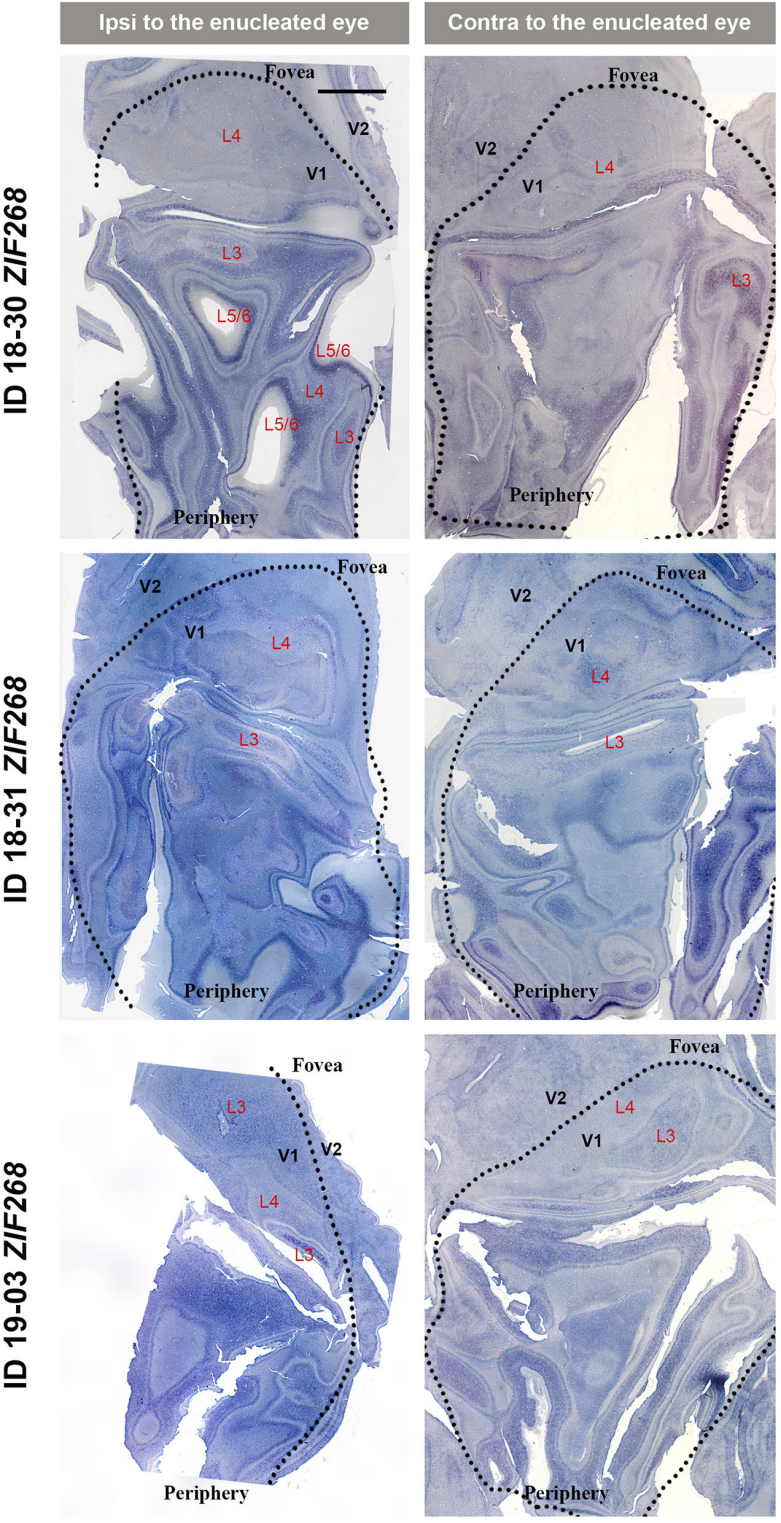
Mosaic images of tangential sections of V1 of eye enucleation cases (ID 18-30, ID 18-31, and ID 19-03) stained for *ZIF268* mRNA. The left panels show the ipsilateral, and the right panels show the contralateral hemispheres to the enucleated eye. It is intended to mainly exhibit layer 4 (4C), but other layers of V1 are also included in each image, illustrated as L3-L6. Black dashed lines delineate borders between V1 and V2. V1 regions representing the foveal and peripheral portions of the visual fields are indicated as “Fovea” and “Periphery,” respectively. Scale bar = 5 mm.

**Figure 4:**
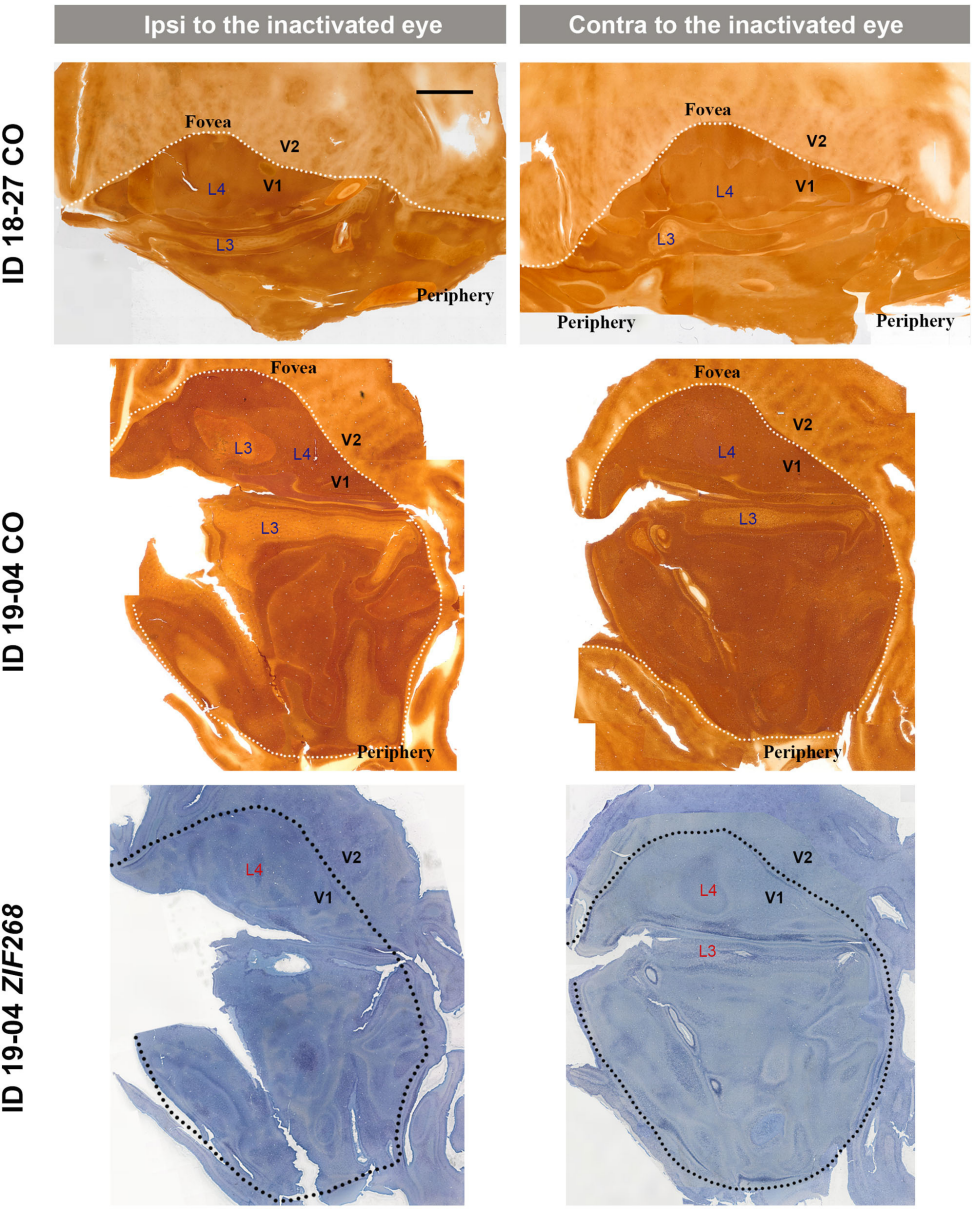
Mosaic images of tangential sections of V1 of TTX injection cases (ID 18-27, and ID 19-04) stained for CO and *ZIF268* mRNA as annotated. The left panels show the ipsilateral, and the right panels show the contralateral hemispheres to the inactivated eye. It is intended to mainly exhibit layer 4 (4C), but other layers of V1 are also included in each image, illustrated as L3–L4. White or black dashed lines delineate borders between V1 and V2. V1 regions representing the foveal and peripheral portions of the visual fields are indicated as “Fovea” and “Periphery,” respectively. Scale bar = 5 mm.

V1s of the monkeys were flattened and stained for CO activity to determine if ODCs were present. V1 was easily discernable due to intense reactivity in layer 4 (4C) and characteristic blobs in layers 2/3 (Carroll and Wong-Riley, 1984). As layer 4 (4C) generally shows the most distinct pattern of ODCs (Adams and Horton, 2003; Takahata et al., 2014), we focused on layer 4 (4C). All sections that included layer 4 (4C) were scanned with the automated microscope and mosaic images were manually made for the whole V1 (Figure 2). However, CO staining pattern was homogeneous throughout V1 and no stripes or patchy patterns were observed in the three monocular enucleation cases. Since IEG expression can reveal maps of cortical neuronal activity more directly than CO histochemistry (Takahata et al., 2014), we applied ISH for *ZIF268* to these V1 sections, and manually made mosaic images for layer 4 (4C) (Figure 3). Again, staining patterns were homogeneous at least in this layer throughout V1. CO reactivity and IEG mRNA expressions were also examined in the monocular TTX cases (Figure 4). Similar to the monocular enucleation cases, staining patterns were homogeneous in layer 4 (4C) throughout V1. These results suggest that these squirrel monkeys lacked or had very minor ODCs in layer 4 (4C) of V1, reconfirming previous observations (Hendrickson and Tigges, 1985; Adams and Horton, 2003).

### IEG Expression in Other Layers

**Figure 5:**
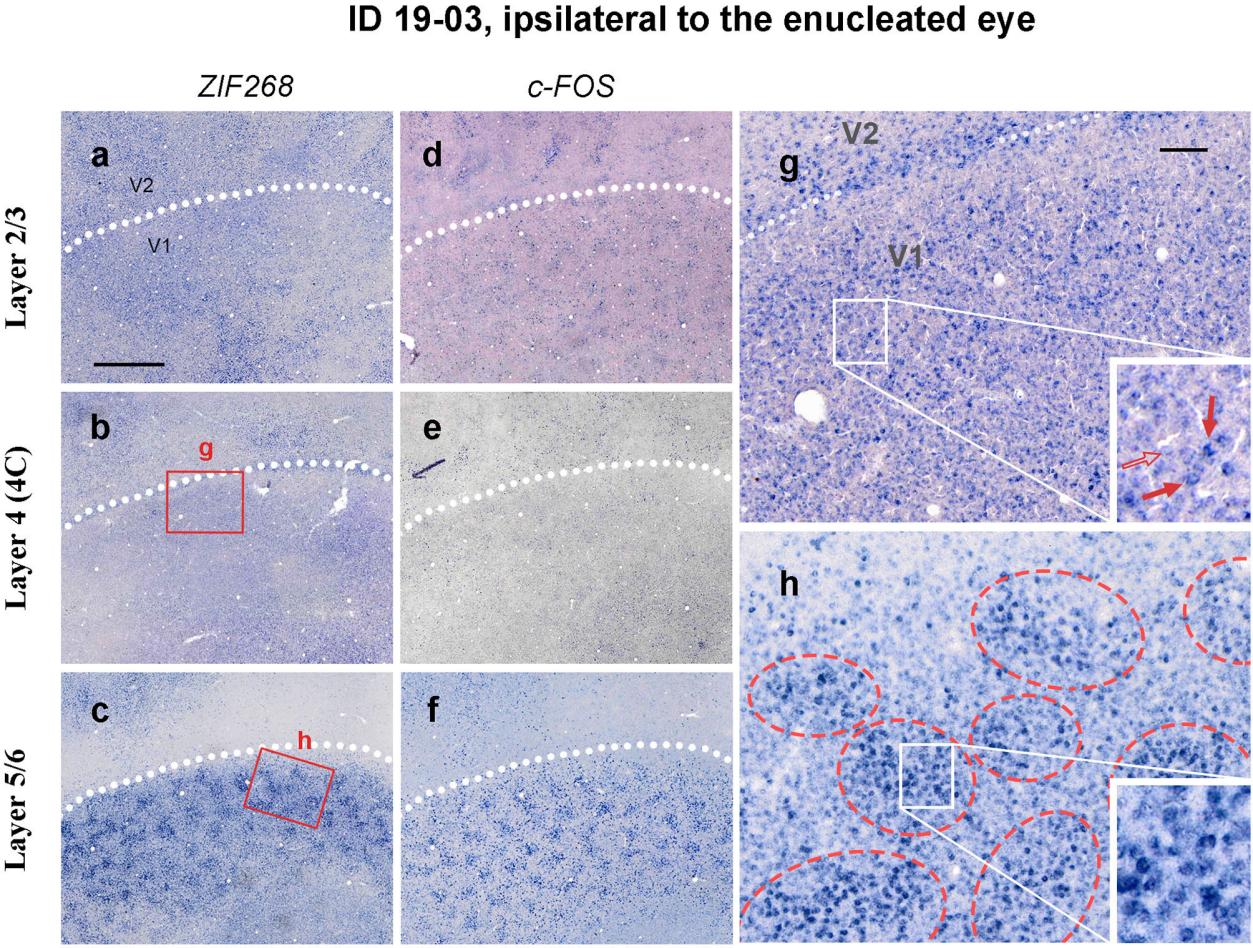
Higher magnification of tangential sections of V1 stained for *ZIF268* **(a–c)** and *c-FOS* **(d–f)** mRNA (ID 19-03, left). Different layers [layer 2/3 for **a,d**, layer 4 (4C) for **b,e**, and layer 5/6 for **c,f**] are compared for a corresponding part. White dashed lines indicate V1/V2 borders. **(g,f)** Higher magnification of the V1 area in b and c (boxed areas). Whereas, the IEG expression only demarcates the V1/V2 boundary in layer 4 due to slightly higher expression levels at the border, it reveals regular patchy compartments in layers 5/6 (indicated by red broken circles). Single-cell level magnification is shown in the insets. Intensely labeled cells are indicated by red arrows, and a lightly labeled cell is indicated by an open arrow. Scale bar in a = 1 mm for **a–f** and scale bar in g = 100 μm for **g** and **h**.

Whereas staining patterns were generally homogeneous in layer 4 (4C), ISH patterns of IEG were patchy in other layers, particularly in layers 5/6 (Figures 5a–f). Staining patterns were similar between ISH for *ZIF268* and ISH for *c-FOS*. At higher magnifications, both intensely labeled cells and lightly labeled cells were observed in each layer (Figures 5g,h). In infragranular layers, however, intensely labeled cells were observed within 150–300 mm diameter clusters, whereas the distribution was rather random in layer 4 (4C).

**Figure 6:**
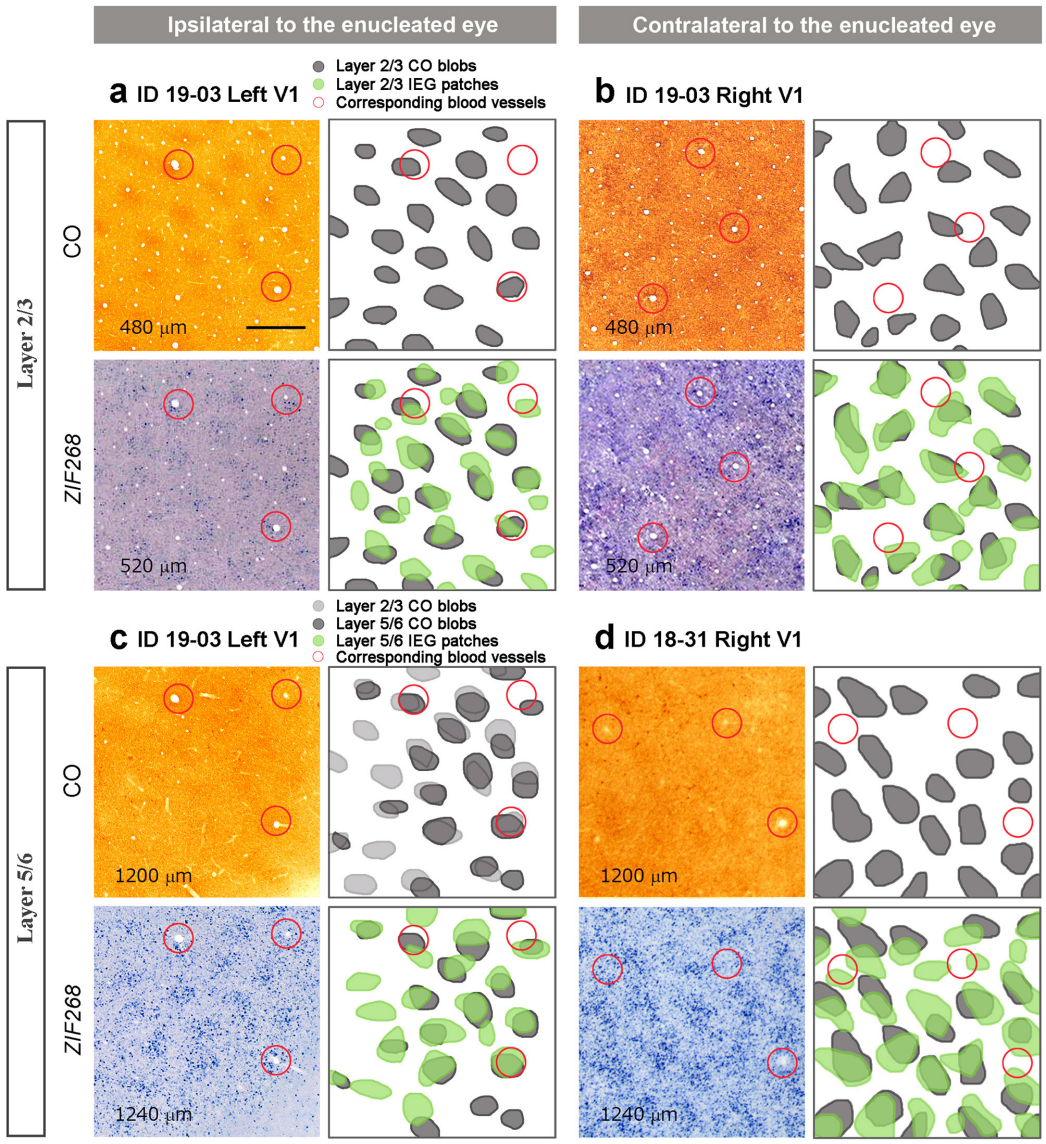
Comparison between CO histochemistry and ISH for *ZIF268* in adjacent tangential sections of V1 (layer, staining, Case ID and hemispheres are as indicated in the figure). **(a)** and **(b)** are in layers 2/3, and **(c)** and **(d)** are in layers 5/6. **(a)** and **(c)** are corresponding V1 parts of the same hemisphere ipsilateral to the enucleated eye, while **(b)** and **(d)** are the V1 contralateral to the enucleated eye from different animals. Depth from the pial surface of each section is provided in each panel. CO blobs and IEG patches are marked and superimposed in the right panels. Corresponding blood vessels between staining are circled in red. Scale bars = 500 μm for all.

We wondered whether the patchy IEG expression outside layer 4 (4C) was related to ocular dominance. On initial inspection, they appeared similar to CO blobs in superficial layers, and thus we hypothesized that CO blob neurons preferentially receive inputs from the ipsilateral eye, and interblob neurons preferentially receive inputs from the contralateral eye as observed in peripheral V1 of owl monkeys (Takahata et al., 2014), or vice versa. If this is true, then CO blobs and IEG patches would be correspondent in one hemisphere and complementary to the other hemisphere, assuming that CO blob patterns are not influenced by MI. To test this possibility, we carefully compared CO blobs and IEG patches with adjacent sections guided by vasculature patterns (Figure 6). Although IEG patches were faint in superficial layers, we were able to confirm that they corresponded to CO blobs in ipsilateral V1 to the inactivated eye (Figure 6a). Different from our hypothesis, IEG patches corresponded to CO blobs in superficial layers of contralateral V1 as well (Figure 6b). In infragranular layers, CO blobs were faint but we were able to confirm that they corresponded to CO blobs in superficial layers. In addition, CO blobs in infragranular layers corresponded to IEG patches in infragranular layers of the V1 ipsilateral to the inactivated eye (Figure 6c). Furthermore, infragranular CO blobs also corresponded to infragranular IEG patches in the contralateral V1 (Figure 6d). Altogether, IEG patches corresponded to CO blobs in both superficial and infragranular layers in both hemispheres, and thus are unlikely to represent ocular dominance patterns of neurons.

**Figure 7:**
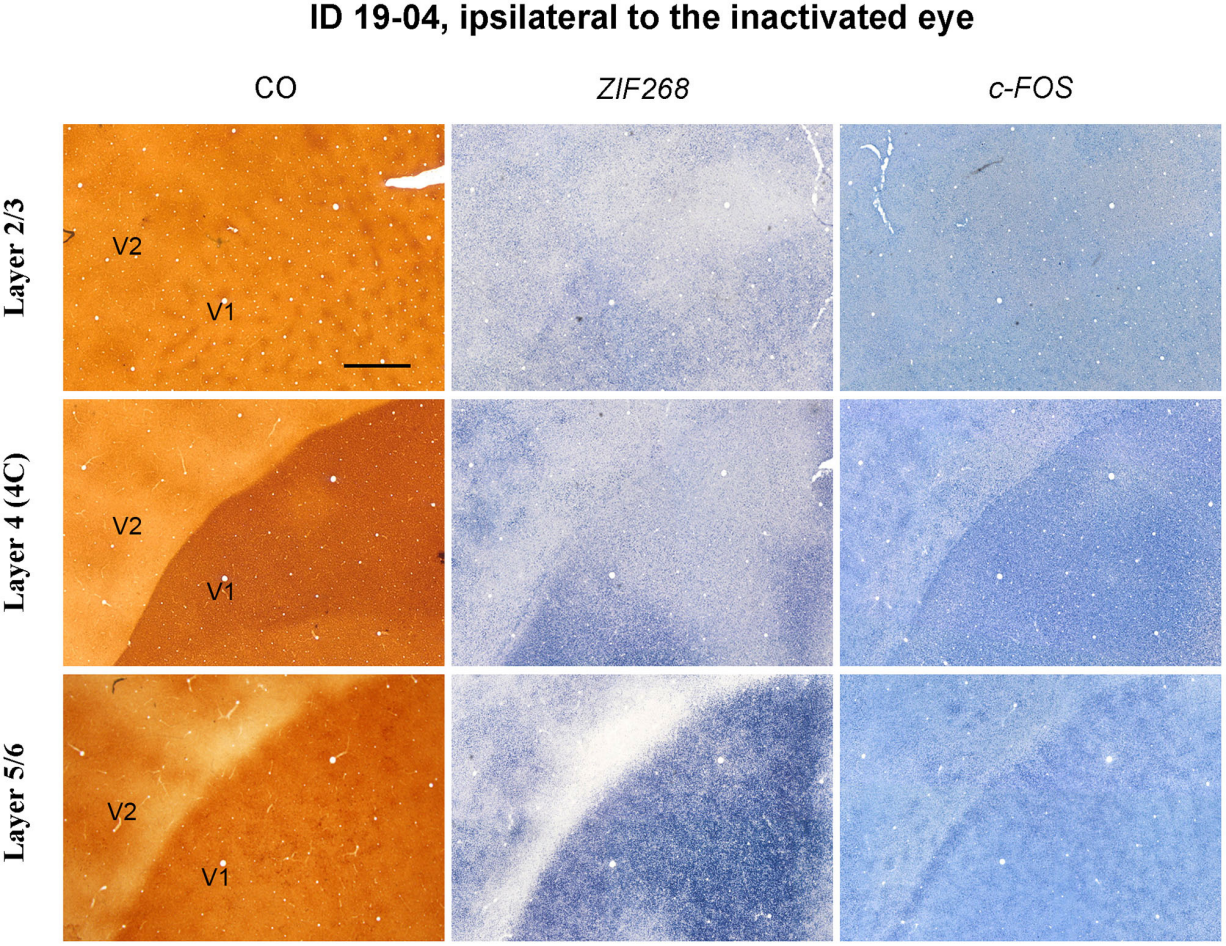
Higher magnification of tangential sections of V1 stained for CO, *ZIF268* mRNA, and *c-FOS* mRNA (ID 19-04, left) as annotated. Different layers are compared for a corresponding part. Scale bar = 1 mm.

CO blob-like IEG patches were also observed in superficial and infragranular layers of V1 in the TTX cases (Figure 7). However, patchy patterns appeared less conspicuous compared to the enucleation cases, implying that IEG patches arise depending on length or strength of MI treatment.

## Discussion

Since “capricious” expression of ODC was reported in squirrel monkeys 18 years ago (Adams and Horton, 2003), no study has re-examined ODCs in this species, whereas many newer studies have made progress in our understanding about ODCs including discoveries of ODCs in other species (Adams et al., 2007; Kaskan et al., 2007; Nakagami et al., 2013; Takahata et al., 2014; Laing et al., 2015; Najafian et al., 2019). Basically, our current results were consistent with previous reports regarding an absence of ODCs in normal adult squirrel monkeys, even though we used more sensitive methods than previously. Even so, distinct patchy patterns were observed coinciding with CO blobs, particularly in infragranular layers. When ODCs were previously detected in squirrel monkeys, there were significant cross-individual variations of ODC representation (Adams and Horton, 2003). However, none of the subjects in this study showed any evidence of ODCs. Perhaps, our subjects were genetically related to each other. If so, ODC representation is not very variable (Kaschube et al., 2003). Nonetheless, squirrel monkeys can be important subjects to study function and development of ODCs.

### Technical Issues

ODCs in V1 had been revealed by various methods: first electrophysiology (Hubel and Wiesel, 1968), then histology, such as transneuronal tracer and CO histochemistry (LeVay et al., 1985; Horton and Hocking, 1998b). In the late 1980s, the uptake of radio-labeled 2-deoxy-D-glucose (2-DG) was measured to map differences in neuronal activity between deprived and non-deprived ODCs (Tootell et al., 1988). More recently, researchers used live imaging techniques, such as functional MRI, intrinsic signal optical imaging, and multi-photon calcium imaging to reveal cortical maps, which not only showed ODCs but also revealed spatial relationships with other functional maps of V1 such as orientation columns (Cheng et al., 2001; Kaskan et al., 2007; Lu and Roe, 2008; Garg et al., 2019). All of these techniques have pros and cons, such as having results restricted to a particular layer, having low spatial resolution and requiring pre-treatment before imaging. A demonstration of ODCs with IEG expression following MI was first introduced in macaques by Chaudhuri et al. (1995) and was followed by its use in other studies (Horton et al., 2000; Soares et al., 2005). Besides macaques, this procedure has been applied to marmosets and cats (Markstahler et al., 1998; Van Der Gucht et al., 2000; Nakagami et al., 2013). The IEG method was also used to reveal architectonic parcellation in the mouse visual cortex (Van der Gucht et al., 2007). Those studies illustrated that IEG expression can imitate CO staining and neurofilament staining, but they did not use IEG expression to thoroughly study the representations of ODCs. We have noticed that the activity-dependent changes in IEG expression are capable of revealing functional compartments that have not been revealed by CO methods in macaque, owl monkeys, tree shrews and even rats (Takahata et al., 2009, 2014; Laing et al., 2015; Takahata and Kaas, 2017). This IEG method enables researchers to study ocular dominance-related structures in all layers of entire V1 and even outside V1 at a single cell-resolution. In addition, availability of specific ISH probe is almost promising for any gene and any animal species, whereas the signal/noise ratio of immunohistochemistry largely relies on availability of specific antibody, which has been commonly used to study IEG expression. Furthermore, light exposure for 1–3 h following dark rearing can peak signal/noise contrast using IEG methods (Nakagami et al., 2013).

Transcription of IEG is triggered by intracellular calcium and cAMP, which are enhanced through the activation of NMDA receptors and L-type calcium channels, and it is thought that the expression is enhanced by neuronal activity where an action potential is coincident with synaptic activity (Bito et al., 1997). Likewise, the expression of IEGs is directly coupled with neuronal activity and the turnover rate of IEG transcripts is remarkably fast; their accumulation peaks at ~30 min after the onset of neuronal activity and decreases to background level ~30 min after the offset of neuronal activity (Zangenehpour and Chaudhuri, 2002). In contrast, it takes a few days of stimulation to reflect activity changes in CO histochemistry (Wong-Riley, 1979; Takahata et al., 2009). An important difference between *c-FOS* and *ZIF268* is the existence of a negative feedback loop for the expression of *c-FOS* which leads to negligible levels after prolonged/repeated stimulations (Sassone-Corsi et al., 1988), but not for *ZIF268*. This suggests that the use of *c-FOS* expression as a mapping tool is more suitable for the detection of transient activity change, while *ZIF268* expression would rather reflect ongoing synaptic activity (Sauvage et al., 2019). Therefore, we believe that examination of IEG mRNA is the most sensitive among currently available methods of revealing ODCs, although it requires to sacrifice animal's life. As a caution, there are some minor differences in their expression during development or in laminar pattern among different IEGs (Mower and Kaplan, 2002; Soares et al., 2005; Sauvage et al., 2019). Besides, transcription of IEG can also be triggered through the activation of receptors for chemical signaling molecules, such as TrkB for BDNF in absence of electrical activity or increase of cytosolic calcium (Pizzorusso et al., 2000). Thus, one needs to be aware of it when he/she evaluates IEG signals as neuronal activity marker in the brain.

### Patchy IEG Expression Outside Layer 4

Our results are basically consistent with the previous idea that there is no ocular dominance segregation at geniculo-striate afferent terminals in a majority of squirrel monkeys (Hendrickson et al., 1978; Hendrickson and Wilson, 1979; Hendrickson and Tigges, 1985; Adams and Horton, 2003). Although an electrophysiological study demonstrated a substantial presence of monocular cells in layer 4 (4C) of V1 in this species (Adams and Horton, 2006), and our observation of scattered neurons with intense IEG labeling may illustrate a distribution of ocular dominance cells in layer 4 (4C), any functional domain was not observed even at a micro architecture level. We observed, however, patchy expression patterns outside layer 4 (4C) particularly in infragranular layers, which corresponded to CO blob patterns. We did not have purely naïve squirrel monkey case in this study, therefore, we cannot show IEG expression patterns without deprivation treatment. However, we argue that the appearance of blob-like patchy patterns is associated with our visual treatments, since the patchy pattern was relatively obscure in TTX cases, and CO blob-like patchy IEG patterns have not been observed in other species without eye treatment. We first thought that the patchy pattern may represent ocular dominance segregation in squirrel monkey V1, but it is unlikely because the IEG expression was high in blobs and low in interblob regions in both hemispheres. The previous electrophysiological and anatomical study also showed no geographical correlation between CO blobs and ODCs, and that neurons located outside layer 4 are highly binocular in squirrel monkeys, different from macaques (Adams and Horton, 2006).

CO histochemistry shows some blob-like patterns in infragranular layers, especially in New World and prosimian primates (Carroll and Wong-Riley, 1984; Condo and Casagrande, 1990), but the pattern is usually faint. In our interpretation, these faint patchy patterns of CO activity represent the distribution of minor afferent inputs from the thalamus (Takahata, 2016; Yao et al., 2021). However, the patchy IEG pattern observed in this study was particularly distinct in infragranular layers and may provide new insights into our understanding of the functional organization of V1. The clear IEG pattern in infragranular blobs is reminiscent of our macaque study (Takahata et al., 2009). In the macaque, the IEG expression level in blobs exceeds surrounding regions, especially in infragranular layers, within a few hours after monocular deprivation or inactivation treatment. In contrast, IEG expression appears homogeneous within the identical ODCs in chronic deprivation (several days to weeks) cases. Other than that, there is no study describing similar to infragranular blob domains. One possible interpretation is that this domain is related to depth perception. Perhaps, interblob neurons in infragranular layers code binocular disparity, and thus decrease their firing rate to less than half in the condition of MI, although such disparity coding neurons are reported to reside in V3 and in thick stripes of V2 in macaques (Chen et al., 2008; Anzai et al., 2011). Alternatively, infragranular blob neurons may code non-disparity depth perception. 3-D depth perception does not only depend on binocular disparity, but also monocular cues, for example, the sense of perspective from blurriness of color, degree of defocus, speed of movement and overlap of objects (Caziot et al., 2017; McCann et al., 2018). Apparently, there are still many unknown circuits related to binocularity and depth perception, and blob-related functional architecture within the primate V1, but it is especially difficult to study these circuits in infragranular layers because optical imaging or multi-photon calcium imaging cannot access these layers. So far, histology is the only available method to study sub-millimeter scale functional architecture in deep layers.

### Significance of Studying ODCs in Squirrel Monkeys

Previous studies of CO expression and the current study of IEG expression report that most squirrel monkeys lack ODCs in normal conditions, despite the fact that ODCs have been reported in a wide variety of species. Especially among primates, ODCs have been clearly observed in New World marmosets, owl monkeys, spider monkeys, capuchin monkeys (Florence et al., 1986; Hess and Edwards, 1987; Rosa et al., 1988; Chappert-Piquemal et al., 2001; Kaskan et al., 2007; Nakagami et al., 2013; Takahata et al., 2014), and even in prosimian galagos (Xu et al., 2005), as well as many genera in Old World monkeys, chimps and humans (Hubel and Wiesel, 1977; Hendrickson et al., 1978; Tigges and Tigges, 1979; Florence and Kaas, 1992; Adams et al., 2007). ODC-like patches have been reported even in rodent V1 (Laing et al., 2015; Andelin et al., 2020). As far as we know, the squirrel monkey is the only exception among primate species. On the other hand, some individuals showed clear ODCs in CO expression (Adams and Horton, 2003, 2006), and transneuronal tracer also exhibited some ocular segregation in geniculo-striate inputs (Horton and Hocking, 1996). More dramatically, large ODCs were clearly observed in a strabismic squirrel monkey, illustrating that ODC formation is facilitated when visual interaction is weak between the two eyes (Livingstone, 1996). Apparently, some minor differences in genetics or visual experience divide the fate of ODC occurrence in squirrel monkey V1. Thus, squirrel monkeys can be good subjects for studying the underlying molecular and cellular mechanisms of development and evolution of ODCs. For example, in squirrel monkeys in which ODCs are observed, ODCs tend to localize in peripheral visual field of V1, and prefer layer 4b (4Cb) rather than layer 4a (4Ca). From this observation, it has been hypothesized that a difference in the relative timing of the maturation of geniculo-striate inputs and intracortical lateral connectivity determines the variability of ODC expression in New World monkeys (Livingstone, 1996). A theoretical neuroscience study suggested that three types of ODC patterns, that are stripe, blob, and uniform, can occur according to the values of the correlation strength and the degree of activity imbalance between the two eyes (Tanaka, 1991). Another group suggests that the balance between the size of afferent sorting filter (receptive field of LGN afferent) and the density of LGN afferents in the binocular zone of V1 determines whether ODCs arise or not (Mazade and Alonso, 2017; Najafian et al., 2019).

As for functional aspects, one study measured visually evoked potentials of a squirrel monkey responding to random-dot stereoscopic stimuli. Contrary to the idea that ODCs serve important roles for stereopsis, V1 neurons of the squirrel monkey showed responses that were consistent with the provided stereoscopic cues, suggesting that the monkey was capable of stereoscopic depth perception (Livingstone et al., 1995) (although please note, only a single monkey was tested and the animal's brain was not examined for the presence of ODCs). In addition, the visual acuity of squirrel monkeys is as good as that of humans (Cavonius and Robbins, 1973; Merigan, 1976). These studies together indicate that the occurrence of ODC formation is not directly required for stereopsis, and led the idea that ODCs are an epiphenomenon of developmental programming (Horton and Adams, 2005). The more direct question is whether the presence of ODCs is necessary for disparity coding neurons to emerge. As this question have been discussed previously (Livingstone et al., 1995; Horton and Adams, 2005; Adams and Horton, 2006), although the regularity of ODCs seems to provide an excellent means of comparing the inputs from the two eyes in an orderly fashion, the squirrel monkeys seem to be able to encode disparity information without their benefit, or at least with only a poorly developed version of them. Perhaps, ODCs are essential for stereopsis in most primates and other animals with binocular overlap, and squirrel monkeys have some quite different and unique mechanism for detecting stereopsis. These monkeys may discriminate depth, for example, by combining information about eye vergence with information about correlation and uncorrelation in the two eyes as discussed above. As another possibility, the distinct and stripe-like character of ODCs may not be necessary for stereopsis, although grouping of cells with similar eye preference would seem likely to facilitate the connections involved. Regardless, our current data urge further study of infragranular neurons of V1 for possible involvement in depth perception as well.

On the other hand, theoretical neuroscientists suggest that cortical neurons increase “wiring economy” by making clusters of synchronizing neurons within a limited space (Mitchison, 1991; Swindale et al., 2000; Koulakov and Chklovskii, 2001; Nauhaus et al., 2016). According to them, several different types of synchronization, such as receptive field, orientation preference, color preference, ON/OFF center preference, as well as ocular dominance in the visual cortex, compete with each other to develop different types of clusters or columns. Finally, they compromise with each other within a columnar space to create a final form that maximizes economy, as represented by the shape of ODCs. The absence of ODCs may illustrate the lack of this competition of cluster formation among different visual features, due to sufficient space or weak orientation preference and/or color preference. Therefore, studying orientation maps and color maps in squirrel monkeys may provide insights into the development of cortical columns.

## Data Availability Statement

The raw data supporting the conclusions of this article will be made available by the authors, without undue reservation.

## Ethics Statement

The animal study was reviewed and approved by The Institutional Animal Care and Use Committee of Zhejiang University.

## Author Contributions

SL and TT designed research and wrote the manuscript. SL, SY, QZ, and TT conducted experiments. All authors contributed to the article and approved the submitted version.

## Funding

This research was supported by National Natural Science Foundation of P. R. China 31872767, 91732305, and 32170992 to TT.

## Conflict of Interest

The authors declare that the research was conducted in the absence of any commercial or financial relationships that could be construed as a potential conflict of interest.

## Publisher's Note

All claims expressed in this article are solely those of the authors and do not necessarily represent those of their affiliated organizations, or those of the publisher, the editors and the reviewers. Any product that may be evaluated in this article, or claim that may be made by its manufacturer, is not guaranteed or endorsed by the publisher.
